# Nonsuicidal self-injury and intersubjective recognition: ‘You can’t argue with wounds’

**DOI:** 10.1177/00380261231221661

**Published:** 2024-02-01

**Authors:** Peter Steggals, Ruth Graham, Steph Lawler

**Affiliations:** Newcastle University, UK; Newcastle University, UK; University of York, UK

**Keywords:** intersubjectivity, nonsuicidal self-injury, recognition, self-harm, stigma

## Abstract

This article explores the relevance of intersubjective recognition and the ‘recognition theoretical turn’ to our understanding of nonsuicidal self-injury. While previous research has demonstrated that self-injury possesses an important social dimension alongside its intrapsychic characteristics, a major challenge for any social approach to self-injury has been to find a way to describe and analyse this dimension without reductively implying that self-injury is a form of ‘attention-seeking’, where this describes a pejorative accusation of social manipulation. One possible solution to this challenge lies in the concept of intersubjective recognition and the idea that what some have interpreted as ‘attention-seeking’ behaviour is perhaps better understood as recognition-seeking. As such, we draw on data from a 2016–2017 English pilot study to examine three basic questions: (1) does self-injury constitute, at least in some cases and amongst its many other observed intrapsychic and social functions, a form of recognition-seeking? (2) if so, how does self-injury work as a claim to recognition? and (3), how do we solve the apparent contradiction of using a stigmatic mark as a means of claiming a normative status? Our study suggests that one of self-injury’s intersubjective imperatives is the need to be listened to and taken seriously, to have one’s feelings and experiences confirmed by others as being legitimate and valid. As such, intersubjective recognition does appear to form a distinct part of the overdetermined complex of meanings and effects associated with self-injury and may be an important factor in a number of cases.

## Introduction

The idea that there is an important relational, and even communicative, dimension to nonsuicidal self-injury^1^ at work alongside its intrapsychic characteristics has often been acknowledged in research (Klonsky & Muehlenkamp, 2007; Muehlenkamp et al., 2012; Nock, 2008; Nock & Prinstein, 2004, 2005; Prinstein et al., 2009; Rodham et al., 2004; Rouski et al., 2021; Steggals et al., 2020a, 2020b, 2022b; Turner et al., 2012). However, this idea has had to proceed cautiously. For many, any suggestion that self-injury is relational is tantamount to dismissing it as ‘attention-seeking’ (Steggals et al., 2020a), where this implies a deceptive performance of distress used to effect a social manipulation and garner positive caring attention from others (Dixon-Ward & Chan, 2021; Issakainen, 2014). And there has certainly been a long and problematic history of such dismissiveness (Chandler, 2016; Pembroke, 1996; Scourfield et al., 2011). As such a major challenge for any social approach to self-injury is to find a way to describe its relational and interactional aspects without reductively implying that they constitute attention-seeking in this pejorative sense.

One possible solution to this challenge is illustrated by the mental health activist Louise Pembroke when she asks:

What is attention-seeking? I know that I do not seek the degradation I have received in Accident and Emergency, neither do I want to be treated with sympathy nor pity . . . But if ‘attention’ means being listened to and taken seriously, then along with the rest of the human race I’m attention-seeking. (1996, pp. 44–45)

What may be interpreted by some as ‘attention-seeking’, then, is perhaps better understood as *recognition-seeking* (Steggals et al., 2020a, p. 163), where *recognition* refers to the basic human need to be listened to and taken seriously by others; to be *acknowledged* as ‘fully human’ (Frank, 1991, p. 87) and *affirmed* in all the normative expectations that this implies (Heidegren, 2004; Honneth, 1995; McBride, 2013). As a participant in Hewitt’s study explains, ‘[s]elf-injury seems to be a great self-destructive attempt to *become human*. To gain *recognition*, to prove to someone that I matter, and that I bleed too’ (1997, p. 57, our emphasis; see also Chandler, 2016, pp. 140–143).

As such, intersubjective recognition may provide a promising alternative to the discarded model of attention-seeking, allowing us to re-address the social dimension of self-injury, promote further social research on this important issue, and contribute to the destigmatization of help-seeking and the social disclosure of self-injury. However, recognition is a contested term (Bankovsky & Le Goff, 2012; Fraser & Honneth, 2003; Honneth & Rancière, 2017) and it is not immediately obvious how the conceptual resources of the ‘recognition theoretical turn’ of the last three decades (Heidegren, 2004; Honneth, 2021; McBride, 2013) could be applied to self-injury. For example, much of this work is more normative than descriptive, and focuses largely on issues of political identity and legal rights, particularly as these apply to the concept of citizenship and to distinct political identities such as those related to race, ethnicity, migration, gender and sexuality. As a result, it has tended to promote an abstract macro-political and legalistic approach to recognition that leaves out everyday life and micro-social processes (Jacobsen, 2010; Jacobsen & Kristiansen, 2009): that is, just those processes most likely to be at work in cases of self-injury and the often intimate face-to-face spaces where it has its greatest social impacts, given that those who self-injure do not constitute or mobilize as a distinct political population. Indeed, self-injury appears quite dissimilar to traditional recognition-seeking strategies such as petitions, protests and lawsuits. In addition, a further challenge lies in the fact that a growing body of research associates self-injury with shame (Gunnarsson, 2021a, 2021b, 2022) and so figures self-injury more as a stigmatic mark of ‘spoiled selfhood’ (Goffman, 1968) than as a means of claiming a normative status (the status of being fully human, of being someone that matters).

In this article we address these issues and draw on empirical data from a 2016–2017 English pilot study to examine three basic questions: (1) can self-injury constitute, amongst its many other observed intrapsychic and social functions, a form of recognition-seeking? (2) if so, how does self-injury work as a claim to recognition? and (3), how do we solve the apparent contradiction of using a stigmatic mark as a means of claiming a normative status? Our study suggests that intersubjective recognition may indeed constitute a factor in the meaning and practice of nonsuicidal self-injury. We find evidence of both self-recognition and social recognition in our data – processes which draw on the symbolic capital of the body to underwrite the self-injuring subject’s claims about their experience, identity and sense of self. It is important to note, however, that there is a broad consensus amongst researchers that self-injury is an overdetermined practice, serving a range of functions which vary between and within cases (Bentley et al., 2014; Hass & Popp, 2006; Hooley & Franklin, 2017; Klonsky & Glenn, 2009; Klonsky and Muehlenkamp, 2007; Lloyd-Richardson et al., 2007). So, while recognition may be a distinct part of this complex, and while it may be an important factor in a number of cases, this *does not* mean that it describes anything like a universal essence of self-injury, or that it will be a factor in *all* cases. Indeed, the extent to which recognition processes are a factor across cases of self-injury, and their distribution relative to other factors, will need to be established by further research working with larger and more diverse samples.

### Methodology

Our data come from a 2016–2017 pilot study concerned with exploring the degree to which close interpersonal relationships shape people’s practices and experiences of self-injury, and how these relationships are themselves affected and shaped by self-injury. Twenty-seven semi-structured, in-depth qualitative interviews were conducted (21 with women and 6 with men) with people who self-injure (*n* = 9), people who are in close interpersonal relationships with people who self-injure (*n* = 12) and people who have had both experiences (*n* = 6). Participants came from a range of class backgrounds; their ages ranged from late teens to early seventies; and while most participants identified as heterosexual, one identified as bisexual.

The interviews explored people’s experiences and understandings of self-injury; their insights into how self-injury may be tied to their interpersonal relationships; and their insights into how self-injury had affected these relationships. No direct questions about recognition were asked, and no particular categories of relationship were targeted in recruitment. The study aimed to better understand the interaction between self-injury and the key relationships forming its immediate social context whether these relationships were familial or friendship relationships. Our analysis here represents one key strand of the findings from the overall study, and other findings have been reported separately (Steggals et al., 2020a, 2020b).

Given that this was a pilot study with a small sample, our findings are necessarily limited and exploratory. Subsequent larger studies may be able to explore a broader range of relationships and therefore generate a broader range of data about how self-injury affects and is affected by close interpersonal relationships. In particular, it ought to be noted that all participants in the study were UK citizens living in England. All participants identified as white-British except one who was born in Austria but who has lived in England for most of her life; one who is British-Indian; and one who identified as being of white-British/Pakistani mixed ethnicity. As such, while our findings may provide interesting suggestions about the role of recognition in self-injury more broadly, they can only speak directly to the cultural contexts described by our participants. Further studies will need to explore the relevance of recognition processes to self-injury in other contexts.

## Recognition theory: A short introduction

Despite its many formulations we might broadly define intersubjective recognition as a ‘basic medium of social integration’ (Heidegren, 2004, p. 365) through which the subject’s identity and sense of self are *acknowledged*, and the normative status that this identity is taken to imply *affirmed*, by a variety of particular and generalized others. Theories of recognition, then, are based on the insight that our sense of identity and selfhood is to some significant degree intersubjectively constituted, and that we only come to understand and evaluate who we really are by the way we are *reflected* in the regard that others hold for us, and the way this regard is articulated through their interactions and relationships with us.^2^ For this reason, recognition is often understood to be a necessary element in the health and freedom of the self while, as Taylor notes, ‘[n]onrecognition or misrecognition can inflict harm, can be a form of oppression, imprisoning someone in a false, distorted, and reduced mode of being’ (Taylor, 1994, p. 25). For Honneth, mis- or withheld recognition means that the subject is unable ‘to count as the person which he [*sic*] desired to be in terms of his Ego ideals’ (Honneth, 1992, p. 199). And Butler powerfully describes misrecognition as something that can ‘undo one’s personhood, undermining the capacity to persevere in a livable life’ (2004, p. 1). As already noted, much recognition theory focuses on issues of political identity and legal rights. While this approach may provide some background to individual cases of self-injury, and the sources of distress that fuel them, it is unlikely to clarify whether self-injury can *in itself* be usefully understood as a strategy of recognition-seeking. This is because self-injury appears to be a meaningful way to ‘experience and express feelings of personal distress and social estrangement’ that is available to various populations across markedly different social situations and contexts (Steggals, 2015, pp. 48, 56). So, while self-injury may be linked to multiple forms of marginalization and misrecognition (Inckle, 2020; Kokaliari & Berzoff, 2008; McDermott & Roen, 2016), it cannot be reduced to any particular form. And, if it is functioning for some as a strategy of recognition-seeking, then it must be doing so at a broader and more generalizable level than the specific forms of estrangement that make up the context of distress in any given case.

However, alongside this macro-political tradition of recognition theory there is also what we might call, adapting a term from Crossley (1996, pp. 15–16, 20), an *egological* tradition, and this may provide a more promising approach. This egological tradition is concerned with intersubjectivity and recognition as foundational, not just for the ongoing dynamics and ethics of selfhood, but for the very *origin*, *structure* and *process* of the ego.^3^ This tradition has its roots in Hegel’s dialectic of self-consciousness (1807/1977). For Hegel, our self-consciousness emerges from the dynamic gap between our ego-ideal and the evidence we can find from the world around us to support this ideal. Though this social mediation in our self-knowledge is necessary (Solomon, 1983, p. 433) it nonetheless creates a tension between the subject’s *dependence* on the world, and its essential inclination to assert its *independence* and autonomy, to be self-sufficient and unconditioned by outside forces. Paradoxically, then, the only way for a subject to have their independence confirmed is to have it affirmed *through their social relationships* (Hegel, 1807/1977, p. 110). However, our need for recognition is mutual, and if *our* self-consciousness depends upon the recognition others will afford us, *their* self-consciousness likewise depends upon the recognition that we may afford them. In this way, recognition emerges as key structure mediating our social relationships: just as we seek recognition from the other, and fear their withholding it, so we may withhold or refuse recognition as a means of asserting our status in relation to them. It is for this reason that Garcin famously declares that ‘Hell is other people’ in Sartre’s *No Exit* (1955) in which three people forced to be roommates in hell slowly come to realize that it is the mutually enforced triangle of recognition sought and recognition withheld that is to be their eternal torment, and not the more traditional tortures that they were expecting.

Hegel’s dialectic of self-consciousness is more eidetic than empirical: however as Honneth (1995) argues, recognition nevertheless remains a key element in psychosocial development. Building on Hegel, Honneth’s theory of recognition also draws on the work of Mead (1934/1967) to provide a naturalistic framework for understanding the emergence of personal subjectivity within an intersubjective matrix; and on psychodynamic object-relations theory, in particular that of Winnicott (1965) and Benjamin (1988), to provide an account of psychological development that rests on the relationship a child has with her primary caregivers (Honneth, 1995, pp. 95–107). Honneth argues that it is our ‘primary relationships’ – our relationships with close family, friends and partners – that are essential to the development and maintenance of ‘self-confidence’, that is:

. . . a very basic sense of the stability and continuity of oneself as a differentiated individual with particular needs and emotions. This basic confidence must also extend to the stability and continuity of the world outside, focused particularly on the social world of significant and anonymous others. (Zurn, 2015, p. 31)

According to Honneth’s use of object-relations theory, the origins of self-confidence lie in the relationship between the infant and her primary caregiver. The infant originally enjoys a kind of symbiosis with its caregiver which provides the context of mutual recognition and emotional support within which both infant and caregiver, in their different ways, can develop into fully individuated beings in relation to one another; and by which each comes to acknowledge the other as a separate body and person with their own desires and needs. The process can be difficult however, and Honneth describes a delicate internal balance between *self-sacrifice* on the one hand and *self-assertion* on the other (1995, pp. 95–107). Both the infant and the caregiver feel the need for individuation, that is, the need to grasp and possess oneself as one’s own person; but this individuation process can only unfold in a healthy way within the context of a strong and supportive emotional bond – a bond which naturally but paradoxically exerts an anti-individuating pull. Consequently, for both Benjamin and Honneth, this process of emerging mutual recognition is best described as a ‘struggle’ (Honneth, 1995, p. 18).

This egological tradition of recognition theory brings us closer to the intersubjective dynamics of self-injury than does the more macro-political tradition that dominates the recognition theoretical turn. This is especially true given that Honneth’s concept of self-confidence refers to not only the *continuity* of a sense of selfhood but also its *stability*: ‘the ability to trust oneself, to be able to be relaxed in the face of the various eruptions and emotions one experiences within oneself, to be able to be alone with oneself without basic anxiety or a sense of the alien character of one’s own needs and drives’ (Zurn, 2015, p. 31). Understood in this way, a lack of ‘self-confidence’ (in Honneth’s sense) may be a common characteristic of self-injury. But as suggestive as this description is, it is not sufficient grounds to assert that self-injury is tied up with recognition and its lack, or to affirm that self-injury itself may constitute a strategy for negotiating recognition. For this we may need a significant theoretical addition to the egological tradition, but first we will need an empirical connection between self-injury and the need for recognition. It is to this that we now turn.

## Is self-injury recognition-seeking?

As already stated, there is a broad consensus amongst researchers that self-injury represents an overdetermined complex of meanings and effects. As such, the question we are exploring here is not whether the need for recognition directly or completely explains self-injury. But rather we are concerned with whether the desire for recognition and the activity of seeking this recognition from self and others, may contribute a single but possibly significant aspect to the overdetermined complex of meanings and effects often associated with self-injury. As Sam, a 21-year-old woman with a history of depression and self-injury, explains, providing a sense of this range of meanings and effects, ‘sometimes it really is just for yourself, a very private, a private pleasure as such erm, [but] I think, no, I think sometimes yeah, you do want other people to understand how you’re feeling and, and, and not necessarily doing it vocally’.

While we are unaware of any research that has explicitly focused on the role of recognition in self-injury, data drawn from both quantitative and qualitative studies suggest that it may play a significant role for some people. For example, in quantitative research, Hawton et al.’s (2006) schools study used a list of eight core motives to elicit respondents’ reasons for ‘self-harm’ (here implying both self-injury and attempted suicide). They found that 40.7% of respondents identified with the motive ‘I wanted someone to know how desperate I was feeling’, while 31.3% identified with the motive ‘I wanted to find out whether someone really loved me’. And in Heath et al.’s (2009) study of university students, 47.8% of respondents identified their motive for self-injury as being ‘to communicate hurting’ while 65.2% identified with ‘social/external motivations’ (e.g. ‘feel close to someone’, ‘get attention’, ‘to not feel like an outsider’) (2009, p. 183).

Within qualitative research, there are numerous direct statements that implicate recognition in people’s understanding of their self-injury. We have already quoted Hewitt’s participant, for example, who describes self-injury as ‘a great self-destructive attempt to become human. To gain recognition, to prove to someone that I matter, and that I bleed too’ (1997, p. 57). In addition, Dawn, a participant in Steggals’ study, discusses her simultaneous desire to both hide and show her self-injury, and describes it as something that exists somewhere between ‘the desire for self-effacement and the desire, not to be looked at exactly, but just to be recognised’ (2015, p. 160). And in our own study, Julie, a 35-year-old support worker reflecting on her experiences of teenage self-injury, reports a lack of recognition from her parents, remarking that she ‘didn’t feel valued as a person’. This led her to develop a ‘secret life’ which included cutting herself with scissors and burning herself on a cooker. She explains:

. . . for me being valued and recognised is pretty much key to any kind of good relationship . . . I think that being recognised and then valued are fundamental to belonging and belonging is something that I have felt the lack of a lot in my life, searching for a sense of belonging, and really liking when I occasionally find it . . . For someone who has spent a lot of time feeling bad or guilty and probably trying to cover up what they are really like, to have someone see you and say, I recognise these things about you (and I like/value them) is a big deal.

Recognition, then, appears as a relevant concern across multiple studies. In our own dataset, recognition appeared as a theme in two primary ways: (1) in statements that framed self-injury as a way to represent or demonstrate the *reality* and *validity* of the self-injuring subject’s inner feelings to themselves and/or others, and therefore demanded recognition for these feelings; and (2) in accounts that tied self-injury to the misrecognition of the self-injuring subject, and their need to have their sense of identity and status acknowledged and affirmed by themselves and/or others. As detailed below, this latter aspect was most typically associated with the change in identity from childhood to early adulthood, and a recourse to self-injury as a strategy for renegotiating the subject’s social bond with their parents.

### Representation and validation

Self-injury is often described as an outward expression of an inner pain (Chandler, 2016, pp. 83–87, 117). For example, Tess, a 32-year-old woman recalling her years of teenage self-injury, notes that she ‘was hurting emotionally but had no physical evidence of pain’. Self-injury gave Tess a way to embody and articulate her feelings. However, the word ‘evidence’ is telling, as if the reality of her suffering was somehow questionable without a physical wound to validate it. Scarry notes that whenever an idea is contested ‘the sheer material factualness of the human body will be borrowed to lend that [idea] the aura of “realness” and “certainty”’ (1985, p. 14). And though Scarry is referring to politics at the societal level, her observation works at the more personal level as well. For example, the musician Richey Edwards famously cut the characters ‘4REAL’ into his forearm in response to an accusation of inauthenticity made by a music journalist (Steggals, 2015, pp. 52–53), providing a challenging confirmation of McLuhan’s famous dictum, albeit raised in a different context, that ‘the medium is the message’ (1964). And as George, a man in his mid-forties with a history of self-injury, explains, ‘[i]t’s like the mental pain, or emotional pain . . . isn’t real but physical pain is. You can’t argue with wounds, they’re there.’

Wounds, then, may provide not only outward testimony to the lived fact of an inward reality, but also a guarantee, written in blood and pain, as to the validity and authenticity of that reality. Wounds are ‘4REAL’. As George explains:

. . . there’s something about the wounds being visible that’s soothing, as if, in some way it’s a visual recognition of the pain, um, and there’s something comforting about it as well, I still, I still when I’m, when I’m acutely stressed I still sometimes think about cutting my wrists, not, not suicidal cutting, but there just seems to be something about it that is a validation of the pain inside, as if the manifestation of it as a visual somehow makes it more real.

This may be one reason why the visual aspect of much self-injury is so often reported as significant (Chandler, 2016, pp. 109–144). As Monica, a woman who began self-injuring at the age of 40 as a result of workplace bullying, states, ‘[t]here was something about having something to show for it, having scars and being able to look at them and, and think “that’s where I’ve been injured”’. Sam agrees, explaining, ‘I need it [self-injury] to represent what I’m feeling . . . if I, you know, if I can’t see, there’s not a proper gout [*sic*], if my skin doesn’t split open, if it’s just a skin wound, if it’s not open and I can’t see anything, then what’s the point?’ As these statements illustrate, the wounds of self-injury appear to provide a kind of validating testimony. However, in these statements, this testimony is largely focused on *self-validation*, a confirmation *to the self-injuring subject themselves* that their pain is indeed deep and genuine. As we have argued elsewhere:

. . . [p]eople who self-injure often question the validity of their inner experience, worrying that it does not describe real pain so much as reflect a narcissistic or histrionic defect of character. Expressing invisible inner pain as a palpably visible bodily wound acts as an emotional release . . . but also . . . a form of validation or authentication. (Steggals *et al.,* 2020a, p. 160)

Nonetheless, our data not only suggest that self-injury may work as an egological *self-recognition*, but also, for some, as a fully *social recognition*. For example, Debra, a 23-year-old woman who started self-injuring at age 12, recalls that she wanted her friend, with whom she had an intense relationship, to be aware of her self-injury:

I think part of me wanted her to see it. Um, everybody else I would rather they didn’t know but for some reason, her, I wanted her to see. I guess because I had this sort of obsession with her in a way. I wanted her to see. But I was hoping as well that it would bring us closer together or, or something or just to get attention from her I guess or, um, yeah, some kind of affection.

In terms that recall Pembroke’s statement quoted earlier, Carol, a woman in her early twenties, reflects that:

. . . sometimes I wonder if I do it to be taken more seriously as someone who’s ill, or more like, ’cus I’m very aware nine times out of ten when you talk to me you wouldn’t know anything was wrong sort of thing . . . and then when you go to the doctors for things I feel like quite often I’ve been dismissed very easily, and sometimes I wonder if one of the reasons I do it, is sometimes to be like ‘no, look: there is a problem. Look! I’m hurting myself’ sort of thing. And like that is attention seeking in a way I suppose but it’s to try and like actually try and get taken seriously as someone who’s ill and needs help.

According to our interpretation of the data, then, the wounds and scars of self-injury, can function as a demand for recognition from both self and others by testifying to and providing evidence of the inner truths of the subject; and by demanding through a recourse to the body that this truth be taken seriously: that it matters, that it is ‘4REAL’.

### Misrecognition and renegotiating social bonds

A second way in which recognition appeared to emerge as a theme in our data was through statements and stories that tied self-injury to the *misrecognition* of the self-injuring subject, and their need to have their own sense of their identity and status acknowledged and affirmed by themselves and/or others. In line with Honneth’s appropriation of object-relations theory, many of these participants reported that these issues first emerged in their transition from childhood to young adulthood.^4^ For example, Sam explains that while she had previously felt intimidated and manipulated by her parents she had also been eager to please them and do well at school; however this changed in her early teens when ‘suddenly all that sort of just started to change and I just started losing [pause] hmmmm, or stopped caring basically.’ It was at this time that she started to self-injure. She first cut herself when her parents grounded her for piercing her ears without their permission. ‘I don’t know if it was revenge’, she explains ‘[but] I felt like “you’re not letting me be who I want to be. I’m an individual, I should be able to do what I want to do.” You know? And er, I think it was like, I felt so upset that no one was listening to me.’ Sam traces her self-injury to a complex of issues but kept returning to the themes of not being taken seriously as an individual and of not having her autonomy respected. Her self-injury appears to be tied to these issues and formed part of a broader pattern of challenging behaviour that communicated to herself and her parents that she was no longer the child she once was, and that her new sense of identity carries new expectations about how her individuality and autonomy should be acknowledged and affirmed.

The role of self-injury in potentially challenging a parent’s sense of their child’s identity, and renegotiating the social bond between child and parent is even more conspicuous in Paula’s story. Paula, a woman in her late forties, recalls the seemingly sudden change in her 14-year-old daughter, Mary, which coincided with a short but intense period of self-injury:

. . . she started shutting herself away in her bedroom. Um, I’d clear the bedroom, I’d find the tissues um, with blood, and that used to bother me, used to make me feel sick. And then I found um, there was like letters and things where she kind of left them out, I think, for me to see . . . and it [sighs], it was really, really difficult because this was in the summer and in the January I’d got a photograph of them stroking horses and she was still in piggy-tails. So it was like one moment I had this little girl and the next moment I had this . . . stranger . . . This really angry stranger.

By rupturing her mother’s expectations of how a young child should act and present herself, Mary forced Paula to reassess her daughter’s identity. Once Paula recognized that her daughter was no longer a child they were able to develop a new relationship and a new pattern of communication, one which Paula credits with helping to stop Mary’s self-injury. Certainly, factors other than her relationship with her parents seemed to have played a role in Mary’s cutting. But it also seems that Mary’s self-injury may have been a part of a renegotiation of the parent–child social bond: a way of compelling a recognition from her parents that she was no longer the little girl with the ‘piggy-tails’.^5^

Our study, then, suggests that processes of both self-recognition and social recognition form a part of the overdetermined complex of meanings and effects associated with self-injury, and may be an important factor in a number of cases. These processes of recognition appear primarily through the self-injuring subject’s felt need to validate their inner world; and through experiences of misrecognition from others, accompanied by the need to attest to the pain of misrecognition, and validate the self-injuring subject’s own sense of their identity. However, if self-injury can be understood (at least in some cases) as a strategy of recognition, then it must be something that is not just motivated by a desire for recognition, but that also *works as a means of eliciting this recognition*. And it is to this issue that we now turn.

## How does self-injury function as a claim to recognition?

According to our interpretation of the data, self-injury can be related to claims of both *self-recognition* and *social recognition*, however it is important to understand that, as Hegel and Honneth make clear, both are *intersubjective* in their essence. Self-recognition works through the internalization of the field of intersubjective interaction (Mead, 1934/1967) such that we can become objects for ourselves (Bartky, 1990, p. 85; Goffman, 1968, p. 18), enter a relationship with ourselves, and thereby make it possible to validate (or invalidate) ourselves, to some degree. For example, Chandler notes in relation to her discussion about the difference between ‘emotional release’ and ‘emotional expression’ that:

. . . expression more directly implicates others – expression invites a *response*. This is more clearly apparent if we consider the concept of invalidation. Emotional expression can be invalidated: while others may respond in a nurturing, affirming or sympathetic manner; equally, they may not respond, and they may turn away or ignore the expression. (2016, p. 83, emphasis in original)

Expression, then, even when it is self-expression, indicates an internalized intersubjectivity that can carry implications about the in/validity of that which is expressed. Consider Tess’s description of what happened after the first time she self-injured:

When I realised I had harmed myself to the point I’d drawn blood I was at first fearful that I’d get into trouble. When time went by and no-one noticed I realised this was a way I could show I was feeling pain without anyone knowing . . . [I] took some comfort in seeing the marks on my arms . . . [they] reminded me that I had inflicted them in response to my feelings about my family and the situation. I think basically it reminded me that I wasn’t going mad, that something was wrong at home and I wasn’t imagining things.

Tess’s statement that self-injury was a ‘way I could show I was feeling pain without anyone knowing’ demonstrates both the objectification of the self and the internalization of the intersubjective field since, at this stage, her self-injury is secret and yet she still feels the need to ‘show’, *to outwardly express*, that she ‘was feeling pain’. Even though she was only communicating *with herself*, she still felt the need to provide evidence *to herself* that her pain was real and not a product of psychosis or imagination. Going one step further, the fact that we can internalize our relationships also means that we can imaginatively interact with both particular and generalized others (Mead, 1934/1967), and so look within for the recognition that we are reluctant to seek from these same others out in the social world. For example, Steggals describes self-injury as being ‘like a letter that has been written but not sent’; addressed to another, yet kept private, always holding out the hope that it may be sent sometime in the future when ‘the recipient will be able to understand it [and] recognise the truths that it contains’ (2015, p. 160). And relatedly, Brossard (2014) talks about ‘virtual opportunities to “seek help”’ (2014, p. 568) where those about to self-injure stage imaginary social scenes in which their intentions are disclosed in the hope of eliciting understanding and even intervention.

Nonetheless, while Steggals notes that the act of writing the letter itself often seems to satisfy ‘the immediate psychological needs of the person self-[injuring]’ (2015, p. 160), the efficacy of this self-recognition necessarily derives from the possibility (and perhaps deferred promise) of a fuller and more genuinely social recognition. As Honneth explains, human subjects ‘can construct and maintain a positive self-relation . . . only with the help of agreeing and affirmative reactions on the part of other subjects’ (2007, p. 143; see also Steggals et al., 2022a). Therefore, our personal claims are never fully established until they are acknowledged and affirmed by others. This suggests that while self-recognition may be an important aspect of self-injury for many people, it may also carry an implicit and deferred desire for a more fully social recognition. For example, Josh, an art-therapist in his twenties, captures this relation between self- and social recognition when he comments that he ‘would make a lot of effort to cover [his self-injury] up, even if there was part of me that wanted someone to see, just so they knew how much I was struggling’.

As Chandler has argued, the secrecy of self-injury:

. . . may be contextualised and not absolute, and there are important reasons why accounts of secrecy may be provided. Individuals may be keen to avoid being charged with ‘attention-seeking’ and therefore may: (a) adapt the management of their scars and wounds and (b) be more inclined to provide accounts which reify secrecy and minimise occasions where self-injury may be ‘less hidden’. (2016, p. 133)

In any case, the letter of self-injury, though kept secret for a while, is in fact often delivered, whether intentionally or not (Heath et al., 2009; Muehlenkamp et al., 2012; Nock, 2008; Nock & Prinstein, 2004, 2005; Steggals et al., 2020a, 2020b; Whitlock et al., 2006). The delivery of this letter means that self-injury subsequently becomes an active communicative fact within a network of relationships: a fact that opens up the possibility of both recognition and misrecognition. But our data strongly suggest that, under these circumstances, it is not the wounds alone that work toward recognition, but the whole complex impact that disclosed self-injury can have in a given social network.

Consider, for example, the letters and blood-soaked tissues that Mary left out knowing that Paula would enter and tidy her room. Similarly, Rachel, a 47-year-old single mother, used to tidy her teenaged daughter’s bedroom and find razor blades and suicide notes. Rachel would make her daughter, Mia, aware that she had found these items, but Mia would continue to leave them suggesting that Mia’s bedroom had become a kind of alternative bulletin board in which things left and things found enabled an alternative mode of indirect communication between mother and daughter. Indeed, those participants who have experienced relationships with people who self-injure typically described a re-framing of their social environment in which the pattern of their interactions, and the *mise-en-scène* of these interactions take on new meaning. Rabindra, whose son Carl has been self-injuring for 11 years, describes how he and his wife, Ingrid, ‘know conditions, circumstances, patterns of behaviour’ that indicate that ‘something is coming . . . We’re quite sort of, your nose gets attuned to it . . . We can pick it up in different ways.’ For example, eating during the night to avoid family dinners, spending more time in his room, and keeping the door to his room resolutely closed, are all signs that indicate to his parents that Carl is struggling and may soon self-injure.

But if self-injury can work as a recognition-seeking strategy through the *social intersubjectivity* of actual social relationships, as well as the *egological intersubjectivity* of self-communication and Brossard’s virtual interaction, then questions remain about how this former kind of recognition can be modelled. Self-recognition can be modelled readily enough by Honneth’s egological recognition theory, but the everyday micro-social spaces and interactions that we have indicated here sit awkwardly between such an approach and the macro-political recognition theory we described earlier (Jacobsen, 2010; Jacobsen & Kristiansen, 2009).^6^ One promising solution to this problem lies, as Jacobsen and Kristiansen (2009) have argued, in Goffman’s concept of the ‘interaction order’: that is, that structure of social rules that are ‘sustained from below’ (Goffman, 1983, p. 6) which shape and regulate both our virtual and our actual co-presence with one another. Goffman provides detailed descriptions of interaction at the micro-social level and, as such, Jacobsen and Kristiansen argue that Goffman’s work on the interaction order can be re-read as providing a micro-social approach to intersubjective recognition (see also Jacobsen, 2010).^7^

Indeed, Goffman provides what might be taken as a basic definition of recognition when he states that: ‘[s]ociety is organized on the principle that an individual who possesses certain social characteristics has a moral right to expect that others will value and treat him [*sic*] in an appropriate way’ (1959, p. 24). And for Goffman this sense of recognition plays out in the intimate and everyday intersubjective spaces where social order is reproduced by a subtle web of interaction rituals. As Jacobsen and Kristiansen explain, Goffman insists on ‘the constitutive, normalizing and stabilizing functions of the many everyday courtesies, the endless stream of respectful interaction, the rule-following behaviour and the recognition of the other as participant in upholding a delicate micro-social order’ (2009, p. 60). In other words, recognition at the micro-social level works through the interaction order. And, as such, any disturbance in this web of everyday courtesies and rule-following behaviours, such as those we have examined here, can carry significant meaning and potentially signal a breakdown in recognition, perhaps forcing others to re-evaluate the situation and the person perceived to be the source of the breakdown The point may be demonstrated dramatically, as with Garfinkel’s infamous ‘breaching experiments’ (1984), but its application to self-injury may direct us toward a more subtle sensibility such as Rabindra and Ingrid’s ‘nose’ for their son’s changing emotional states as these are signalled through closed doors and midnight snacks.

Goffman’s description of the interaction order, then, provides a missing realm of intersubjective recognition active between the egological and the macro-political realms, and as such represents a promising model for further research on self-injury as a potential strategy of social recognition.

## How can a stigmatic mark work as a claim to recognition?

Sociological work on self-injury frequently ties it to Goffman’s (1968) concept of stigma and the experience of a ‘spoiled’ sense of selfhood where the subject fails, or expects to fail, to find ‘acceptance’ from others (Goffman, 1968, p. 19; cf. Tyler & Slater, 2018). Gunnarsson, for example, has argued that shame and self-injury ‘reproduce and amplify each other’ cultivating a difficult and ‘self-perpetuating cycle of shame and self-injury’ (2021a, p. 316; see also Gunnarsson, 2021b, 2022). She notes that, ‘[t]he constitution of a failed self and negative self-image is closely related to how individuals who self-injure, in their interior dialogues with imagined others, conceive and anticipate how others will think and react to them’ (2021a, p. 329). However, while this description affirms the connection between shame, misrecognition and self-injury, it also raises the question of how a stigmatic mark representing a sense of spoiled selfhood can also function as a claim to recognition. In this final section, we draw on theory related to both recognition and self-injury to propose an answer to this question.^8^

Of course, psychodynamic theory has long observed that people’s inner life can be characterized by forms of complex dissonance and ambivalence (Vogler, 2000); and, furthermore, individual cases should be understood within their own dense sociocultural contexts. However, one possible solution to the tension between feelings of shame and the need for recognition might be drawn from Brossard’s (2018) sociological re-framing of the affect regulation model of self-injury. According to this dominant psychological model, ‘unprocessed’ affect can build up within the subject like steam in a pressure-cooker; self-injury functions as an emergency vent, releasing emotional pressure and intervening against a catastrophic eruption. Brossard however argues that much of this emotional pressure represents the *individualization* of a fundamentally social tension. Within ‘specific social configurations’ that direct people to ‘manag[e] their emotions discretely’ (Brossard & Steggals, 2020, p. 217), the self-injuring subject essentially channels this social tension through themselves in order to prevent an embarrassing eruption of normatively unprocessed emotion; this being a threat to the interaction order, the social bonds this order regulates, and the subject’s social presentation of a normative self (see also Chandler, 2016, pp. 85, 90; Gunnarsson, 2021b, p. 117). And as Gunnarsson notes, the internalization and individualization of social tension is often experienced as shame (2021a, 2021b).

It is possible then, to the degree that the subject consciously or unconsciously understands the role they are playing in preserving the interaction order within specific social networks, that they may feel this role characterizes a misrecognition. That is, they may feel that their individualization of a social tension represents an unfair burden, indicating that others within the social network value them and their status less than, or differently to, their perception of what is normative or deserved. Under such circumstances, self-injury may take on dual meanings: on the one hand representing the internalization and individualization of social tension, while on the other hand representing evidence of this individualization and the suffering that it has caused; that is to say, evidence of a *misrecognition*, and the basis therefore for a counterclaim of appropriate recognition.

This dual meaning of self-injury also makes sense in relation to Honneth’s egological theory of recognition. Any experience of ‘moral injury’ (Honneth, 2007) resulting from the withdrawal or refusal of recognition will likely have an inherently ambivalent quality to it as it will be conditioned by our need and desire for recognition on the one hand, and our intersubjective dependence on others for our self-understanding on the other (Honneth, 1995, p. 131). While the former factor may lead us to seek recognition, the latter may lead us to internalize the misrecognition of others, thus creating an intrapsychic ambivalence in which we feel both aggrieved at the moral injury inflicted upon us *and* anxious that this injury may in fact be justified. By extension then, the symbols of moral injury may also be patterned in this ambivalent sense, acting at once as both symbolic claims to recognition *and* as stigmatic marks indicating a spoiled selfhood. And while this dynamic of recognition *claimed* and misrecognition *internalized* may or may not be basic to human (inter)subjectivity, it is arguably a dynamic that has been powerfully staged by ‘Western’ modernity as the core psychosocial drama of personal identity and selfhood. Our need to claim recognition may be amplified through what Durkheim (1893/2013) calls the ‘cult of the individual’ and in particular its discourses of authentic selfhood and the primacy of our inner, emotional truths (Taylor, 2007, p. 475). Our tendency to internalize and individualize misrecognition may be amplified through what Foucault (1982) describes as the *governmental* structure of the subject which locates the individual as the site of a sociogenic micro-power (Martin & Waring, 2018).

## Conclusion

Brossard and Chandler (2022) note that a key challenge for sociological analyses of mental health issues is the need to theorize socially complex phenomena without being reductive. A recognition-based conceptualization of self-injury offers a productive way of deepening our understandings of such complexity. Our study suggests that one of the social factors and intersubjective imperatives that stand behind self-injury is the need to be listened to and taken seriously, to have one’s feelings and experiences confirmed by others as being legitimate and valid. In other words, intersubjective recognition does appear to form a distinct part of the overdetermined complex of meanings and effects associated with self-injury and, as such, it may constitute an important factor in a number of cases – though this does not mean *all* cases, or even a majority of cases. This includes both the egological intersubjectivity of self-recognition, and the thoroughly social intersubjectivity of social recognition. From our analysis, these processes of recognition appear to draw on the visceral immediacy and ‘material factualness’ of the human body (Scarry, 1985, p. 14) to underwrite and evidence the self-injuring subject’s claims of real pain and suffering; and to protest, and even renegotiate, a felt misrecognition of their identity and status.

While our study affirms the association of recognition and self-injury, the strength and distribution of this association will need to be further established by research that draws on larger and more diverse samples, and that, unlike the current study, takes intersubjective recognition as its primary focus. Furthermore, since the processes of social recognition that we have identified here largely play out in the interaction order described by Goffman, future research into self-injury as a recognition practice may benefit greatly from adopting an approach that reads Goffman’s work as a theory of micro-social recognition.

Understanding that self-injury can be recognition-seeking rather than attention-seeking, not only advances our understanding of self-injury and its impact on everyday social contexts and close personal relationships, it also has the potential to help destigmatize, and so encourage, help-seeking and the disclosure of self-injury. Ultimately, a recognition-centred approach emphasizes the fact that we cannot completely separate out the personal from the social, the individual from their community. Our need for recognition means that we can only be free and independent subjects because we are also, and at the same time, *social and relational subjects*: an ‘“I” that is “We” and “We” that is “I”’ as Hegel puts it (1807/1977, p. 110). In our analysis, self-injury represents a disorder of the estranged and isolated ‘I’ in need of the recognition of the ‘We’, whether internalized as self-recognition or fully realized as social recognition. On this view, what the self-injuring subject needs is to be acknowledged and affirmed as fully human, as someone who matters, as someone who also bleeds.

## Notes

1. That is, the typically habitual act of causing direct harm to the body, where the purpose of the act is this harm itself rather than, for example, body modification or suicide.

2. Much recognition theory makes universal claims. Whether these claims are justified, or whether recognition is a more culturally relative phenomenon, is an important question. However, we do not have space to host this debate here. We simply note that our study is UK-based and so the application of recognition theory to our data does not rest on universal claims.

3. Crossley takes this term from Husserl and uses it to differentiate between self-reflexive intersubjectivity and a more ‘radical’ unreflexive intersubjectivity that operates eidetically prior to a sense of ego or self. Here we intend the term to refer to those processes of intersubjectivity that describe the psychosocial development of the subject and the internalization of the social field into the subject’s psychological economy and sense of self.

4. This transition emerged as a theme in the data. It was not specifically asked about or targeted in sampling.

5. Of course, Paula’s account is partial and located and may well differ from Mary’s. But this is true of all such accounts, including others cited here.

6. While Honneth’s theory of recognition is explicitly intersubjective, he models intersubjectivity in an eidetic rather than empirical fashion.

7. Butler’s (2004) work on recognition provides some important theoretical foundations for this observation, as they acknowledge the embodiment of recognition processes in performative acts of social interaction. However, Butler does not empirically map the field of recognition behaviours at the micro-social level and so provides fewer potential tools for empirical analysis than Goffman.

8. It should be noted that some self-injury may be used to lay claim to a ‘spoiled identity’. It has arguably been used this way in punk sub-culture (Steggals, 2015, p. 55). And one of our participants, Carol, stated that she self-injures in order to show others that she is a ‘bad person’ and to ‘warn’ them off.
